# Risk Factors for Corneal Endothelial Cell Loss in Patients with Pseudoexfoliation Syndrome

**DOI:** 10.1038/s41598-020-64126-w

**Published:** 2020-04-29

**Authors:** Takanori Aoki, Koji Kitazawa, Tsutomu Inatomi, Natsuki Kusada, Noriko Horiuchi, Kazunori Takeda, Norihiko Yokoi, Shigeru Kinoshita, Chie Sotozono

**Affiliations:** 1 Department of Ophthalmology, Kyoto Prefectural University of Medicine, Kyoto, Japan; 2 Department of Ophthalmology, North Medical Centre Kyoto Prefectural University of Medicine, Kyoto, Japan; 3 Department of Ophthalmology, Saiseikai Shiga Hospital, Shiga, Japan; 4 Department of Frontier Medical Science and Technology for Ophthalmology, Kyoto Prefectural University of Medicine, Kyoto, Japan

**Keywords:** Eye diseases, Corneal diseases

## Abstract

This study investigated corneal endothelial cell density (ECD) in pseudoexfoliation (PEX) syndrome patients and evaluated the clinical factors associated with ECD for 51 eyes of 41 phakic patients with pseudoexfoliation (PEX group) and 201 eyes of 117 patients with age-related cataracts (control group) as an age-matched control to the PEX group. Variable clinical factors, including ECD, central corneal thickness (CCT), anterior chamber depth (ACD), number of anti-glaucoma eye drops and severity of PEX, were examined using multivariate analyses. Severity of PEX was as follows: Mild in 28 eyes, Moderate in 16 eyes, and Severe in 7 eyes. The mean ECD was 2,548 ± 409 cells/mm^2^ in the PEX group and 2,757 ± 282 cells/mm^2^ in the control group, respectively, and ECD in the PEX group was significantly lower than that in the control group (*P* = 0.02). Multivariate analyses revealed that the severity of PEX [−176.8, 95% confidence interval (CI) (−244.5, −109.2), *P* < 0.01] was significantly associated with lower ECD. Accumulation of PEX materials contributed to early corneal endothelial decompensation.

## Introduction

Pseudoexfoliation (PEX) syndrome was first reported by Lindeberg in 1917, and it was characterized by material like dandruff on the lens and the pupil edge^1^. It was found that PEX materials accumulated not only various ocular tissues but also human body^2^. It has been reported that involvement of PEX materials in the anterior segment of the eye developed open-angle glaucoma, cataract with phacodonesis and corneal endothelial cell loss^3,4^. Many studies in regard to the association between corneal endothelial cell density (ECD) and PEX have shown that ECD was lower in patients with PEX than in normal subjects, that ECD in PEX glaucoma (PEXG) patients was similar to that in PEX patients without glaucoma, and that there was no significant difference between unilateral PEX eye and contralateral eye^5–8^.

Naumann and Schlötzer-Schrehardt proposed a distinctive PEX keratopathy, characterized by diffuse irregular thickening of Descemet’s membrane and marked phagocytosis of melanin pigment by attenuated endothelial cells differentiated from Fuchs endothelial corneal dystrophy (FECD)^9^, which is a major cause of bullous keratopathy^10^. However, PEX keratopathy has been often misdiagnosed as non-guttate FECD^11^. Histopathological studies have revealed that there were focal deposits of PEX material on the posterior corneal surface and in Descemet’s membrane, which appear to be produced by corneal endothelial cells^3^, and they suggest that PEX material may cause endothelial cell decompensation. A small-scale national survey in Japan has reported that the number of bullous keratopathy caused by PEX keratopathy has been increasing^12^.

PEX eyes comprise many abnormalities of the anterior segment. However, few reports have been documented the association between severity of PEX materials on the iris and clinical findings. Recent development of imaging system made it possible to offer us more precise diagnosis, and artificial intelligence (AI) technology would accelerate to develop the efficacy of diagnostic imaging^13,14^. We classified the severity of PEX as four-step grading (None, Mild, Moderate, Severe) based on slit-lamp imaging, and aimed to examine ECD in PEX syndrome patients in comparison to normal subjects and to investigate the association between clinical factors and lower ECD in PEX syndrome patients.

## Results

**Figure 1:**
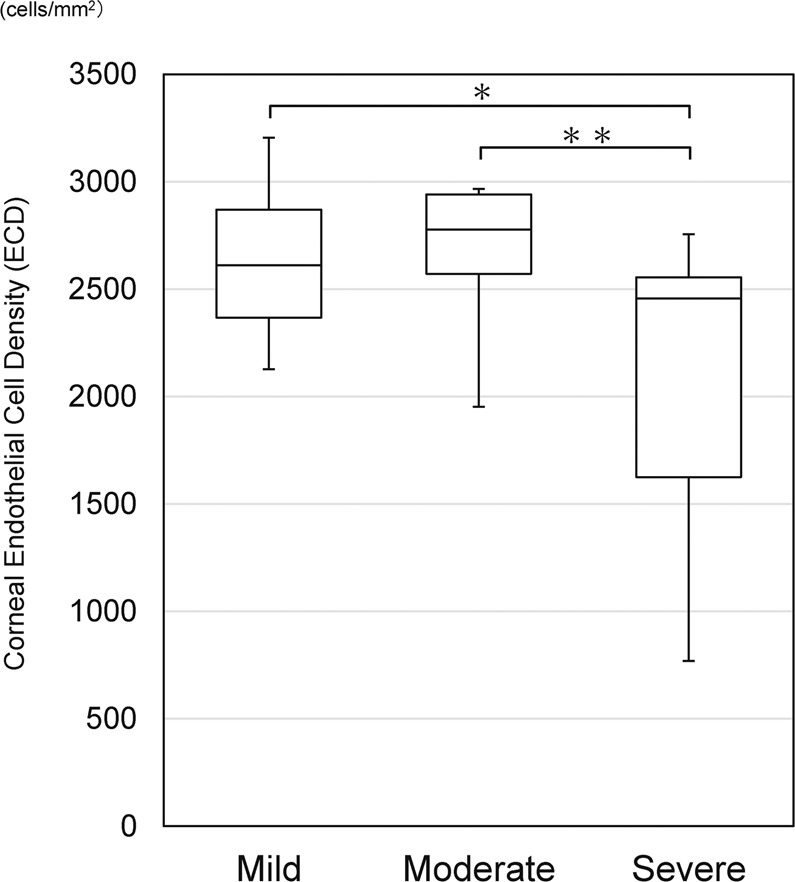
Corneal endothelial cell density (ECD) in patients with mild, moderate and severe PEX, respectively.

**Figure 2:**
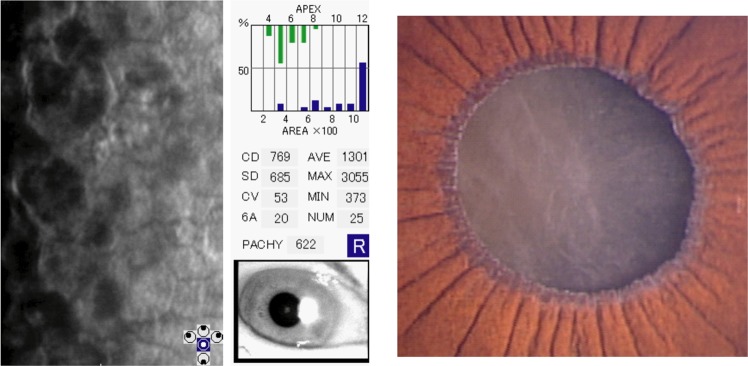
Representative severe pseudoexfoliation (PEX) syndrome patient was an 89-year-old woman with low endothelial cell density (ECD).

The retrospective study included 51 eyes of 41 patients in the PEX group and 201 eyes of 117 patients in the control group. The demographic data, including baseline characteristics of the participants, severity of PEX, the presence of glaucoma, ECD, grading for corneal endothelial damage, CCT, ACD and number of anti-glaucoma eye drops, are shown in Table 1. Severity of PEX in the PEX group was Mild for 28 eyes, Moderate for 16 eyes and Severe for 7 eyes, and severity in the control group was None for 201 eyes. The mean age was 78.7 ± 6.8 years old (range: 60–89) in the PEX group and 77.3 ± 7.3 years old (range: 59–96) in the control group and there was no statistical difference in the age distribution (*P* = 0.60). Glaucoma was involved in 17 of 51 eyes (33.3%) in the PEX group and 18 of 201 eyes (9.0%); primary open angle glaucoma for 10 eyes and normal-tension glaucoma for 8 eyes) in the control group (*P* = 0.001). The mean ECD was 2,548 ± 409 cells/mm^2^ in the PEX group and 2,752 ± 282 cells/mm^2^ in the control group, respectively, and ECD in the PEX group was significantly lower than in that in the control group (*P* = 0.02). Grading for corneal endothelial damage in the PEX group was Normal for  48 eyes (94.1%), Grade 1 for 2 eyes (3.9%) and Grade 2 for 1 eye (2.0%), and grading in the control group was Normal for 199 eyes (99.0%) and Grade 1 for 2 eyes (1.0%). The mean CCT in eyes in the PEX group (534 ± 39 μm) was comparable to that in the control group (530 ± 37 μm) (*P* = 0.54). There was no statistical significance between ACD in eyes in the PEX group (2.9 ± 0.4 mm) and in the control group (3.0 ± 0.4 mm) (*P* = 0.20). Number of anti-glaucoma eye drops in eyes in the PEX group (0.7 ± 1.2 eye drops) was more than in the control group (0.2 ± 0.8 eye drops) (*P* < 0.01). Anti-glaucoma eye drops used in the PEX group and the control group are as follows; prostaglandin analogues for 14 eyes, β-blocker for 11 eyes, carbonic anhydrase inhibitor for 4 eyes, α2 adrenergic agonist for 4 eyes and Rho-kinase inhibitors for 4 eyes in the PEX group, and prostaglandin analogues for 16 eyes, β-blocker for 6 eyes, carbonic anhydrase inhibitor for 4 eyes, α2 adrenergic agonist for 6 eyes and Rho-kinase inhibitors for 7 eyes in the control group. The mean ECD was 2,629 ± 291 cells/mm^2^ in patients with mild PEX, 2,623 ± 198 cells/mm^2^ in moderate PEX, and 2,049 ± 693 cells/mm^2^ in severe PEX, respectively, and ECD in patients with severe PEX was significantly lower than that in mild and moderate PEX *(P* < 0.01, *P* < 0.01, respectively) (Fig. 1). Representative case with severe PEX was shown in Fig. 2.

**Table 1:** Demographics of pseudoexfoliation (PEX) syndrome patients and normal subjects.

	PEX	Control	*P* values
No. of Eyes	51	201	
Age (years old)	78.7 ± 6.8	77.3 ± 7.3	0.60
Gender (male : female)	20 : 31	68 : 133	0.47
Severity of PEX			
None	0	201	
Mild	28	0	
Moderate	16	0	
Severe	7	0	
Glaucoma	17	18	0.001*
ECD (cells/mm^2^)	2,548 ± 409	2,752 ± 282	0.02*
Grading for corneal endothelial damage			
Normal	48	199	
Grade 1	2	2	
Grade 2	1	0	
CCT (μm)	534 ± 39	530 ± 37	0.54
ACD (mm)	2.9 ± 0.4	3.0 ± 0.4	0.20
No. of anti-glaucoma eye drops	0.7 ± 1.2	0.2 ± 0.8	0.001*

ECD: endothelial cell density, CCT: central corneal thickness, ACD: anterior chamber depth, *indicates significant difference.

The univariate analyses of factors that affect lower ECD (age, gender, CCT, number of anti-glaucoma eye drops, severity of PEX) revealed that severity of PEX [−149.5, 95% confidence interval (CI) (−203.9, −95.1), *P* < 0.01] was significantly associated with lower ECD. Multivariate analyses revealed that severity of PEX [−176.8, 95% CI (−244.5, −109.2), *P* < 0.01] was significantly associated with low ECD (Table 2).

**Table 2:** Risk factors of endothelial cell loss in patients with pseudoexfoliation.

Factors	Results	Univariate analysis			Multivariate analysis		
		β	95% CI	*P* Value	β	95% CI	*P* Value
Age (years)	77.7 ± 7.2	1.8	-4.0, 7.6	0.54	2.9	-2.7, 8.5	0.30
Gender (male / female)	88/164	2.2	-40.9, 45.3	0.92	-12.1	-55.0, 30.7	0.60
Severity of PEX		-149.5	-203.9, -95.1	<0.01*	-176.8	-244.5, -109.2	<0.01*
None	201						
Mild	28						
Moderate	16						
Severe	7						
CCT (μm)	524 ± 68	0.3	-0.3, 0.9	0.32	0.3	-0.2, 0.9	0.26
ACD (mm)	3.1 ± 1.9	-4.7	-27.4, 18.1	0.69	-8.1	-29.5, 13.3	0.46
No. of anti-glaucoma eye drops	0.3 ± 0.9	-66.0	-110.6, 21.5	< 0.01*	-25.3	-72.1, 21.5	0.29

β: regression coefficient, CI: confidence interval, PEX: pseudoexfoliation, CCT: central corneal thickness, ACD: anterior chamber depth, *indicates significant difference.

## Discussion

In this present study, our findings found statistically significant lower ECD in PEX patients compared to normal subjects, although there was no guttate finding in the PEX patients. In addition, the univariate and multivariate analyses revealed that severe PEX syndrome indicating more PEX material accumulations on the iris surface was significantly associated with lower ECD. Populations in this present study did not include pseudophakic eyes or eyes with post-intraocular surgery, suggesting that PEX material may cause corneal endothelial cell loss.

It was previously reported by Zimmermann and associates that lower ECD was found with increasing PEX stage, with or without glaucoma. They classified the severity of PEX as two groups (mild and severe) according to slit-lamp microscopy images and glaucoma stage^15^. Naumann and Schlötzer-Schrehardt proposed the stage of PEX syndrome from stage 1 to stage 5 on the basis of histopathologic evidence^9^, which showed abnormal formation of endothelial cells by accumulation of PEX materials, be called PEX keratopathy, finally leading to bullous keratopathy. PEX materials on the corneal endothelial cells were directly observed by *in vivo* confocal microscopy (IVCM) in PEX eyes as the hyperreflective deposits^12^. We classified severity of PEX based on PEX material deposits on the iris surface. Multivariate analysis revealed that severe PEX depositions were significantly associated with lower ECD, suggesting that PEX material accumulation progressed PEX keratopathy, resulting in corneal endothelial decompensation.

In addition, multivariate analysis revealed that more glaucoma eye drops were required in patients with severe PEX accumulation, suggesting that more amount of PEX may be involved in elevation of intraocular pressure, resulting in glaucoma progression, as which is consistent with previous reports that showed the association between PEX and glaucoma^6–8^. There still may be an ongoing debate on whether there was significant difference between ECD in PEX syndrome patients and PEXG patients. Most previous reports suggested that PEX populations with or without the presence of glaucoma lead to an early corneal endothelial decompensation^3,16^. Although anti-glaucoma eye drops, especially benzalkonium chloride (BAK) and prostaglandin analogues, are strong toxic to corneal endothelial cells *in vitro*, the toxicity decreases significantly after 1000-fold or more dilution^17,18^, and is expected be weaken on *in vivo* corneal endothelium. However, elevation of IOP, involvement of BAK and anti-glaucoma eye drops may accentuate the endothelial damage, and further investigation is needed.

Recent imaging system has been now dramatically developing, and there is a potential for future diagnosis system with the integration of AI technology. Many reports have already documented that artificially intelligent diagnosis algorithm for skin cancer and age-related macular degeneration was feasible, and it reached out the same level as a human dermatologist and a retinal specialist^13,14^. Our findings revealed that the severe PEX materials on the iris surface found to be a significant risk factors for corneal endothelial cells loss and glaucoma, suggesting that PEX materials on the iris surface would be effective for early diagnosis and management of anterior segment abnormalities.

It should be noted that this study included some limitations. Our present study was a retrospective study, and the number of patients included in this study was limited. However, all patients with PEX syndrome still had phakic eyes, suggesting that the surgical intervention owing to cataract surgery was minimized and we were able to investigate the direct relationship between PEX and ECD. Another limitation was that we only examined the association between PEX materials on the iris surface and ECD and the patients did not receive the pupil dilatation examination. We are currently planning to investigate the direct relationship between PEX accumulation on the corneal endothelium and ECD using IVCM.

In conclusion, our retrospective and cross-sectional study revealed that PEX material accumulation developed more severe PEX keratopathy, which might result in bullous keratopathy, regardless of the presence or absence of glaucoma. Even after successful corneal transplantation in patients with severe PEX keratopathy, careful attention should be paid to monitor postoperative ECD during long-term follow-up.

## Patients and Methods

This was a retrospective, cross-sectional study conducted from April 2018 to November 2018 at North Medical Center, Kyoto Prefectural University of Medicine, Kyoto, Japan. The study was approved by the review board in Kyoto Prefectural University of Medicine (#ERB-C-1006) and was conducted in adherence with the tenets of the Declaration of Helsinki. Written informed consent was obtained from all subjects without the waiver.

**Figure 3:**
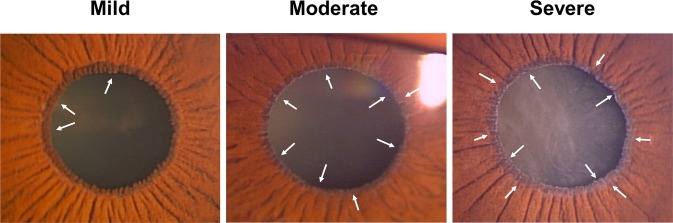
Grading score of pseudoexfoliation (PEX). Severity of PEX was scored based on slit-lamp examination as follows: None, Mild, Moderate and Severe. The arrows on figures show PEX materials.

Two groups of subjects were investigated in this study as follows: (1) PEX syndrome eyes with phakia (PEX group) and (2) age-matched cataract eyes as normal control (control group). Additionally, PEX group and control group were classified into 4 groups based on the amount of PEX materials on iris surface by two ophthalmologists (T.A., K.K.): None for no PEX materials, Mild for PEX materials on part of the pupillary border, Moderate for PEX materials on the entire pupillary border, and Severe for PEX materials on the entire pupillary border and the iris surface (Fig. 3). PEX materials on the naturally pupillary border and iris surface were photographed by slit lamp imaging camera (THD-341, Ikegami Tsushinki Co., Ltd., Tokyo, Japan) and were analyzed with a method modified with a previous report^15^. Inclusion criteria for all study participants were as follows: (1) age above 55 years; (2) no history of laser or intraocular surgery, including glaucoma surgery; and (3) no past history except cataract and open-angle glaucoma.

Corneal endothelial cell density (ECD), grading for corneal endothelial damage (defined by the grading score of ECD according to the Japanese Corneal Society^19^), central corneal thickness (CCT), anterior chamber depth (ACD), number of anti-glaucoma eye drops and severity of PEX were evaluated. All study participants underwent specular microscopy (Noncon Robo 2, Konan, Hyogo, Japan), non-contact partial coherence laser interferometry (IOL Master, Carl Zeiss Meditec AG, Jena, Germany) and optical coherence tomography (DRI OCT Triton, Topcon, Tokyo, Japan). The software marked the central corneal endothelium within 0.24 × 0.4 mm range, then counted the number of corneal endothelial cells and automatically calculated ECD. In some cases which were difficulty in the automated method, ECD was calculated ECD with the manual method. Grading for corneal endothelial damage defined by the Japanese Corneal Society was as follows: Normal for ECD over 2000 cells/mm^2^ or more, Grade 1 for ECD between 1000 and 2000 cells/mm^2^, Grade 2 for ECD between 500 and 1000 cells/mm^2^, Grade 3 for ECD less than 500 cells/mm^2^ without corneal oedema, and Grade 4 for bullous keratopathy. CCT was measured by two individuals by anterior segment optical coherence tomography (AS-OCT), which was DRI OCT Triton with anterior segment attachment. ACD was measured by the IOL Master. Factors that could affect lower ECD (age, gender, CCT, ACD, number of anti-glaucoma eye drops, severity of PEX) were examined using univariate and multivariate analysis.

### Statistical analysis

Statistical analyses were performed using JMP Pro ver. 14.0.0 statistics software (SAS, Cary, NC, USA). To identify risk factors associated with lower ECD in patients with PEX, analyses were performed with univariate and multivariate logistic regression that included age, gender, CCT, number of anti-glaucoma eye drops and severity of PEX as covariates. The incidence of regression coefficient with 95% confidence intervals (CIs) was calculated. A P-value of less than 0.05 was considered statistically significant.

## References

[1] JG, L. Kliniska Undersökningar över Depigmentering av Pupillarranden och Genomlysbarheten av Iris vid Fall av Åldersstarr samt i Normala Ögon hos Gamla Personer. *Thesis Helsingfors* (1917).

[2] Naumann GO, Schlotzer-Schrehardt U, Kuchle M (1998). Pseudoexfoliation syndrome for the comprehensive ophthalmologist. Intraocular and systemic manifestations. Ophthalmology.

[3] Schlotzer-Schrehardt UM, Dorfler S, Naumann GO (1993). Corneal endothelial involvement in pseudoexfoliation syndrome. Arch. Ophthalmol..

[4] Streeten BW, Li ZY, Wallace RN, Eagle RC, Keshgegian AA (1992). Pseudoexfoliative fibrillopathy in visceral organs of a patient with pseudoexfoliation syndrome. Arch. Ophthalmol..

[5] Miyake K, Matsuda M, Inaba M (1989). Corneal Endothelial Changes in Pseudoexfoliation Syndrome. Am. J. Ophthalmol..

[6] Inoue K, Okugawa K, Oshika T, Amano S (2003). Morphological study of corneal endothelium and corneal thickness in pseudoexfoliation syndrome. Jpn. J. Ophthalmol..

[7] Wang M, Sun W, Ying L, Dong XG (2012). Corneal endothelial cell density and morphology in Chinese patients with pseudoexfoliation syndrome. Int. J. Ophthalmol..

[8] Palko JR, Qi O, Sheybani A (2017). Corneal Alterations Associated with Pseudoexfoliation Syndrome and Glaucoma: A Literature Review. J. Ophthalmic Vis. Res..

[9] Naumann GO, Schlotzer-Schrehardt U (2000). Keratopathy in pseudoexfoliation syndrome as a cause of corneal endothelial decompensation: a clinicopathologic study. Ophthalmology.

[10] Adamis AP, Filatov V, Tripathi BJ, Tripathi RC (1993). Fuchs’ endothelial dystrophy of the cornea. Surv. Ophthalmol..

[11] Abbott RL, Fine BS, Webster RG, Paglen PG, Spencer WH (1981). Specular microscopic and histologic observations in nonguttate corneal endothelial degeneration. Ophthalmology.

[12] Zheng X (2013). New findings for an old disease: morphological studies on pseudoexfoliation syndrome-related keratopathy and binocular asymmetry. Cornea.

[13] De Fauw J (2018). Clinically applicable deep learning for diagnosis and referral in retinal disease. Nat. Med..

[14] Esteva A (2017). Dermatologist-level classification of skin cancer with deep neural networks. Nature.

[15] Zimmermann N, Wunscher M, Schlotzer-Schrehardt U, Erb C (2014). [Corneal endothelial cell density and its correlation with the severity of pseudoexfoliation]. Klin. Monbl Augenheilkd..

[16] Sihota R, Lakshmaiah NC, Titiyal JS, Dada T, Agarwal HC (2003). Corneal endothelial status in the subtypes of primary angle closure glaucoma. Clin. Exp. Ophthalmol..

[17] Ayaki M, Iwasawa A, Inoue Y (2010). Toxicity of antiglaucoma drugs with and without benzalkonium chloride to cultured human corneal endothelial cells. Clin. Ophthalmol..

[18] Ayaki M (2009). [Cytotoxicity of antiglaucoma ophthalmic solutions for human corneal endothelial cells]. Nippon. Ganka Gakkai Zasshi.

[19] Kinoshita S (2014). Grading for corneal endothelial damage. Nippon. Ganka Gakkai Zasshi.

