# Publisher Correction: Risk Factors for Corneal Endothelial Cell Loss in Patients with Pseudoexfoliation Syndrome

**DOI:** 10.1038/s41598-020-66089-4

**Published:** 2020-05-26

**Authors:** Takanori Aoki, Koji Kitazawa, Tsutomu Inatomi, Natsuki Kusada, Noriko Horiuchi, Kazunori Takeda, Norihiko Yokoi, Shigeru Kinoshita, Chie Sotozono

**Affiliations:** 1 Department of Ophthalmology, Kyoto Prefectural University of Medicine, Kyoto, Japan; 2 Department of Ophthalmology, North Medical Centre Kyoto Prefectural University of Medicine, Kyoto, Japan; 3 Department of Ophthalmology, Saiseikai Shiga Hospital, Shiga, Japan; 4 Department of Frontier Medical Science and Technology for Ophthalmology, Kyoto Prefectural University of Medicine, Kyoto, Japan

Correction to: *Scientific Reports* 10.1038/s41598-020-64126-w, published online 29 April 2020

This Article contains an incorrect version of Figure 3. The correct Figure 3 appears below as Figure 1.

**Figure 1:**
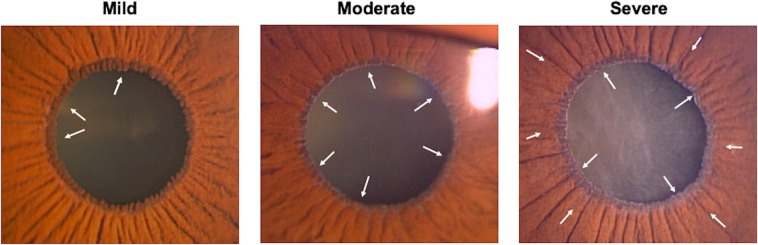
.

